# Accuracy of Influenza *ICD-10* Diagnosis Codes in Identifying Influenza Illness in Children

**DOI:** 10.1001/jamanetworkopen.2024.8255

**Published:** 2024-04-24

**Authors:** James W. Antoon, Tess Stopczynski, Justin Z. Amarin, Laura S. Stewart, Julie A. Boom, Leila C. Sahni, Marian G. Michaels, John V. Williams, Janet A. Englund, Eileen J. Klein, Mary A. Staat, Elizabeth P. Schlaudecker, Rangaraj Selvarangan, Jennifer E. Schuster, Geoffrey A. Weinberg, Peter G. Szilagyi, Ariana Perez, Heidi L. Moline, Andrew J. Spieker, Carlos G. Grijalva, Samantha M. Olson, Natasha B. Halasa

**Affiliations:** 1 Department of Pediatrics, Vanderbilt University Medical Center, Nashville, Tennessee; 2 Department of Biostatistics, Vanderbilt University Medical Center, Nashville, Tennessee; 3 Department of Pediatrics, Baylor College of Medicine and Texas Children’s Hospital, Houston, Texas; 4 Department of Pediatrics, UPMC Children’s Hospital of Pittsburgh, University of Pittsburgh School of Medicine, Pittsburgh, Pennsylvania; 5 Department of Pediatrics, Seattle Children’s Hospital, Seattle, Washington, Washington; 6 Department of Pediatrics, Cincinnati Children’s Hospital Medical Center, University of Cincinnati College of Medicine, Cincinnati, Ohio; 7 Department of Pediatrics, Children’s Mercy–Kansas City, Kansas City, Missouri; 8 Department of Pediatrics, University of Rochester School of Medicine and Dentistry, Rochester, New York; 9 Department of Pediatrics, UCLA Mattel Children’s Hospital, Los Angeles, California; 10 Influenza Division, National Center for Immunization and Respiratory Diseases, Centers for Disease Control and Prevention, Atlanta, Georgia; 11 Department of Health Policy, Vanderbilt University Medical Center, Nashville, Tennessee

## Abstract

**Importance:**

Studies of influenza in children commonly rely on coded diagnoses, yet the ability of *International Classification of Diseases, Ninth Revision* codes to identify influenza in the emergency department (ED) and hospital is highly variable. The accuracy of newer *International Statistical Classification of Diseases and Related Health Problems, Tenth Revision (ICD-10)* codes to identify influenza in children is unknown.

**Objective:**

To determine the accuracy of *ICD-10* influenza discharge diagnosis codes in the pediatric ED and inpatient settings.

**Design, Setting, and Participants:**

Children younger than 18 years presenting to the ED or inpatient settings with fever and/or respiratory symptoms at 7 US pediatric medical centers affiliated with the Centers for Disease Control and Prevention–sponsored New Vaccine Surveillance Network from December 1, 2016, to March 31, 2020, were included in this cohort study. Nasal and/or throat swabs were collected for research molecular testing for influenza, regardless of clinical testing. Data, including *ICD-10* discharge diagnoses and clinical testing for influenza, were obtained through medical record review. Data analysis was performed in August 2023.

**Main Outcomes and Measures:**

The accuracy of *ICD-10–*coded discharge diagnoses was characterized using molecular clinical or research laboratory test results as reference. Measures included sensitivity, specificity, positive predictive value (PPV), and negative predictive value (NPV). Estimates were stratified by setting (ED vs inpatient) and age (0-1, 2-4, and 5-17 years).

**Results:**

A total of 16 867 children in the ED (median [IQR] age, 2.0 [0.0-4.0] years; 9304 boys [55.2%]) and 17 060 inpatients (median [IQR] age, 1.0 [0.0-4.0] years; 9798 boys [57.4%]) were included. In the ED, *ICD-10* influenza diagnoses were highly specific (98.0%; 95% CI, 97.8%-98.3%), with high PPV (88.6%; 95% CI, 88.0%-89.2%) and high NPV (85.9%; 95% CI, 85.3%-86.6%), but sensitivity was lower (48.6%; 95% CI, 47.6%-49.5%). Among inpatients, specificity was 98.2% (95% CI, 98.0%-98.5%), PPV was 82.8% (95% CI, 82.1%-83.5%), sensitivity was 70.7% (95% CI, 69.8%-71.5%), and NPV was 96.5% (95% CI, 96.2%-96.9%). Accuracy of *ICD-10* diagnoses varied by patient age, influenza season definition, time between disease onset and testing, and clinical setting.

**Conclusions and Relevance:**

In this large cohort study, influenza *ICD-10* discharge diagnoses were highly specific but moderately sensitive in identifying laboratory-confirmed influenza; the accuracy of influenza diagnoses varied by clinical and epidemiological factors. In the ED and inpatient settings, an *ICD-10* diagnosis likely represents a true-positive influenza case.

## Key Points

**Question:**

How accurate are *International Statistical Classification of Diseases and Related Health Problems, Tenth Revision (ICD-10)* discharge diagnosis codes in identifying pediatric influenza illness?

**Findings:**

In this cohort study of 32 968 children seeking care for fever and/or respiratory symptoms in the emergency department (ED) and inpatient settings, *ICD-10* influenza discharge diagnosis codes were highly specific, with high positive predictive value and negative predictive value but modest sensitivity compared with laboratory-confirmed influenza. When stratified by age, specificity was lowest among infants.

**Meaning:**

These findings suggest that *ICD-10* influenza diagnoses likely represent true-positive influenza cases in the ED and inpatient settings, but their sensitivity in identifying cases of influenza illness is modest.

## Teaser

This cohort study examines the accuracy of *International Statistical Classification of Diseases and Related Health Problems, Tenth Revision (ICD-10)* influenza discharge diagnosis codes in the pediatric emergency department and inpatient settings.

## Introduction

Influenza virus is a frequent cause of respiratory illness and a leading cause of emergency department (ED) visits and hospitalizations in the US.^1,2^ Observational studies of influenza often use resources such as administrative claims and clinical databases, including electronic health records. These studies typically use coded diagnoses to identify relevant influenza-related events.^3,4,5,6,7,8,9,10^ However, the accuracy of the previously used *International Classification of Diseases, Ninth Revision (ICD-9)* diagnosis codes for identifying influenza in pediatric EDs is poorly characterized. Results from studies of influenza *ICD-9* codes were primarily in the inpatient settings and were highly variable. For example, among hospitalized US children, the sensitivity of an *ICD-9 *influenza diagnosis compared with laboratory-confirmed influenza ranged from 65% to 82%, and the positive predictive value (PPV) ranged from 55% to 88% during the months of October to April and varied by influenza season and study site.^11,12^

In October 2015, health care institutions in the US converted to *International Statistical Classification of Diseases and Related Health Problems, Tenth Revision (ICD-10)* diagnosis codes. As a result, influenza *ICD-10* diagnosis codes are now commonly used to identify influenza in clinical studies, including recent high-profile publications.^3,4,5,6,13^ However, the accuracy of *ICD-10* codes to identify influenza in children in the US is unknown. A better understanding of the accuracy of influenza *ICD-10* codes is needed to evaluate potential misclassification in studies using these codes to identify influenza as either an exposure or an outcome. The objective of this study was to determine the sensitivity, specificity, PPV, and negative predictive value (NPV) of influenza *ICD-10* codes compared with laboratory-confirmed influenza virus. Although most prior studies have focused on the inpatient setting, the ED accounts for a large proportion of health care utilization.^14^ Therefore, we evaluated the accuracy of influenza diagnosis codes in both the ED and inpatient settings.

## Methods

### Study Design and Population

Data from the Centers for Disease Control and Prevention–funded New Vaccine Surveillance Network (NVSN) were used for this study. NVSN is a prospective, active, population-based surveillance network that enrolls children younger than 18 years with acute respiratory illness.^15^ Eligible children include those presenting to the ED or admitted to the inpatient setting with fever and/or cough, earache, nasal congestion, runny nose, sore throat, posttussive vomiting, wheezing, shortness of breath and/or rapid or shallow breathing, myalgia, apnea, apparent life-threatening event, or brief resolved unexplained event of up to 14 days duration at 1 of 7 pediatric medical institutions: Cincinnati Children’s Hospital Medical Center, University of Cincinnati College of Medicine (Cincinnati, Ohio); Baylor College of Medicine and Texas Children’s Hospital (Houston, Texas); Children’s Mercy–Kansas City (Kansas City, Missouri); Vanderbilt University Medical Center (Nashville, Tennessee); UPMC Children’s Hospital of Pittsburgh, University of Pittsburgh School of Medicine (Pittsburgh, Pennsylvania); University of Rochester School of Medicine and Dentistry (Rochester, New York); and Seattle Children’s Hospital (Seattle, Washington). This analysis included data from December 1, 2016, through March 31, 2020, and was performed in August 2023. Influenza season for the primary analysis was defined as the period spanning the first week with 2 or more positive influenza research tests through the last week with 2 or more positive influenza research tests for each surveillance site (referred to as NVSN influenza season).^1,16^ We excluded children with fever and neutropenia associated with malignant neoplasm, revisits within 4 days (when the initial visit was included), or transferred from another hospital after an admission of more than 48 hours. Written informed consent for participation from the parent or guardian and assent, when applicable, were obtained. The institutional review boards at the Centers for Disease Control and Prevention and each of the 7 sites reviewed and approved this cohort study. This report followed the Strengthening the Reporting of Observational Studies in Epidemiology (STROBE) reporting guidelines.^17^

Information about race and ethnicity was captured from the parent and/or guardian during enrollment. If these data were not collected during the interview, race and ethnicity from the medical record was used. These data were collected because care seeking behavior and influenza testing may vary by race and/or ethnicity.

### Influenza Diagnosis Definition

Influenza diagnosis was defined by the presence or absence of an influenza *ICD-10* discharge diagnosis code (eAppendix in Supplement 1).^18^ Influenza diagnosis positive (*ICD-10* positive) was defined as the presence of an influenza *ICD-10* code among individuals discharged from the ED or influenza *ICD-10* code among hospitalized individuals. Influenza diagnosis negative (*ICD-10* negative) was defined as the absence of an influenza *ICD-10* discharge diagnosis code. Individuals admitted to the hospital from the ED were included as part of the inpatient cohort.

### Reference Standard for Influenza Cases

The reference standard to determine the presence of influenza virus is a positive laboratory test. Two forms of laboratory tests were included in this study: research and clinical testing. For research testing, midturbinate nasal swabs with or without oropharyngeal swabs were collected by study personnel from enrolled children. For intubated patients, tracheal aspirates were accepted as alternatives to midturbinate nasal swabs. When midturbinate nasal swabs with or without oropharyngeal or tracheal aspirate specimens were not available, salvaged clinical respiratory specimens were obtained for research testing. Research specimens underwent testing at each site by commercial or institution-specific in-house reverse transcription–polymerase chain reaction (RT-PCR) assays for influenza virus (eAppendix in Supplement 1).^19,20^

Clinical (nonresearch) testing modalities as part of routine clinical care were collected from the participant’s medical records and included the following clinical test types ordered from any setting: (1) rapid antigen, (2) rapid RT-PCR (rapid PCR), and (3) RT-PCR panel (PCR panel) testing. Influenza-positive cases were defined as those with a positive molecular assay, including either a positive PCR molecular research (RT-PCR) and/or clinical (rapid PCR or PCR panel) test. Influenza-negative cases were defined as having a negative research and clinical influenza molecular test. If a patient had a positive rapid antigen test but negative molecular test, they were classified as influenza negative.

### Measures of Accuracy

True-positive cases were defined as those with an influenza *ICD-10* diagnosis code (*ICD-10* positive) and an influenza-positive molecular test (influenza positive). False-negative cases were defined as influenza positive, but without an influenza *ICD-10* diagnosis code (*ICD-10 *negative). Accordingly, false-positive cases were defined as those with an influenza-negative molecular test (influenza negative), but with an influenza *ICD-10* diagnosis code (*ICD-10* positive), and true-negative cases were *ICD-10 *negative and influenza negative.

### Statistical Analysis

Descriptive statistics are presented as median (IQR) for continuous variables and as frequencies (percentages) for categorical variables. We computed the sensitivity, specificity, PPV, and NPV stratified by setting to determine the accuracy of *ICD-10* coding for influenza, with corresponding Wald-based 95% CIs for each influenza season and overall. Sensitivity was calculated as the number of true-positive cases (influenza positive and *ICD-10* positive) divided by all influenza-positive cases. Specificity was calculated as the number of true-negative cases (influenza negative and *ICD-10 *negative) divided by all influenza-negative cases. PPV was calculated as the number of true-positive cases (influenza positive and *ICD-10* positive) divided by all *ICD-10*–positive cases. NPV was calculated as the number of true-negative cases (influenza negative and *ICD-10 *negative) divided by all *ICD-10–*negative cases. Estimates were computed overall and stratified by setting (ED and inpatient) and age (0-1, 2-4, and 5-17 years). All analyses were performed using R statistical software version 4.1.1 (R Project for Statistical Computing).

We performed 6 sensitivity analyses to evaluate the robustness of our findings. First, to comprehensively examine the potential impact of influenza prevalence on our estimates, we evaluated our accuracy measures using 3 commonly used influenza season and influenza peak period definitions: (1) October 1 through March 31 (October to March, broader and more variable by geographic location than NVSN definition), (2) the 13 consecutive weeks that contained the maximum number of influenza cases (ie, peak 13 weeks, typically includes shorter duration but higher prevalence than NVSN definition), and (3) period of time of cases where the onset of symptoms occurred after the date corresponding to the 33rd percentile and before or on the date corresponding to the 67th percentile of symptom onset date among enrolled influenza-positive cases (peak 33%, typically includes highest prevalence weeks compared with NVSN and peak 13 weeks definition). To demonstrate the association of prevalence with seasonal definitions, PPV and NPV for the weeks outside of the peak 13 weeks and peak 33% definitions, but included in the primary NVSN definitions, were reported. We defined peripeak 13 weeks as the weeks of the NSVN season that do not contain the 13 consecutive weeks that contained the maximum number of influenza cases. Prepeak 33% was defined as the weeks where symptom onset occurred before or on the date corresponding to the 33rd percentile of symptom onset date among influenza-positive cases. Postpeak 33% were defined as the weeks where onset of symptoms occurred after the date corresponding to the 67th percentile of symptom onset date among enrolled influenza-positive cases.

Second, PCR has greater sensitivity than rapid antigen testing but is less commonly used in the ED because of its longer result time. Therefore, differences between rapid and PCR testing performance may impact coding practices. To differentiate the impact of testing method (antigen vs PCR) on diagnostic accuracy, we excluded patients with rapid antigen testing from the population. Third, rapid testing (rapid antigen or rapid PCR) generates test results faster than traditional PCR panel testing. To evaluate the association of timing of testing results with diagnostic accuracy, we performed a sensitivity analysis excluding patients with both rapid antigen and rapid PCR testing. Fourth, there were 136 cases with a positive antigen test and negative research PCR test. Given the high specificity of both tests, it is possible that these cases are true-positive influenza cases and were misclassified as influenza-negative cases. We performed a sensitivity analysis evaluating the effect of defining influenza cases as any positive laboratory test (rapid antigen or PCR). Fifth, the sensitivity of PCR tests substantially decreases after 5 days from the time of symptom onset. To evaluate the impact of time from disease onset and time of influenza testing, we performed a sensitivity analysis stratifying individuals up to 5 days and greater than 5 days between the start of symptoms and laboratory testing. Finally, we performed an analysis restricting the definition of a true case of influenza to a positive clinical antigen or PCR test.

## Results

### Study Population

A total of 16 867 children in the ED (median [IQR] age, 2.0 [0.0-4.0] years; 9304 boys [55.2%]) and 17 060 hospitalized children (median [IQR] age, 1.0 [0.0-4.0] years; 9798 boys [57.4%]) were included in the study (eFigure in Supplement 1). Among children in the ED, data on race and ethnicity were available for 16 667 of them: 4342 (26.1%) were Hispanic, 7506 (45.0%) were non-Hispanic Black, 3484 (20.9%) were non-Hispanic White, and 1335 (8.0%) were non-Hispanic other (ie, American Indian or Alaska Native, Asian, Native Hawaiian or Other Pacific Islander, multiple, and none, consolidated because of small sample sizes) (Table 1). Among children who were inpatients, data on race and ethnicity were available for 16 911 of them: 4035 (23.9%) were Hispanic, 4096 (24.2%) were non-Hispanic Black, 7078 (41.9%) were non-Hispanic White, and 1702 (10.1%) were non-Hispanic other (Table 2). Additional details on race and ethnicity are shown in the eAppendix in Supplement 1.

**Table 1.** Demographic and Clinical Characteristics of Patients in the Emergency Department

Characteristic	Patients, No. (%)^a^				
	Total (N = 16 867)	True positive (n = 1193)^b^	False negative (n = 1300)^c^	False positive (n = 168)^d^	True negative (n = 14 206)^e^
Age at presentation, median (IQR), y	2.0 (0.0-4.0)	3.0 (1.0-6.0)	3.0 (1.0-6.0)	2.0 (1.0-6.0)	1.0 (0.0-4.0)
Age range at presentation, y					
0-1	8276 (49.1)	351 (29.4)	392 (30.2)	68 (40.5)	7465 (52.5)
2-4	4911 (29.1)	435 (36.5)	480 (36.9)	48 (28.6)	3948 (27.8)
5-17	3680 (21.8)	407 (34.1)	428 (32.9)	52 (31.0)	2793 (19.7)
Sex					
Male	9304 (55.2)	644 (54.0)	707 (54.4)	85 (50.6)	7868 (55.4)
Female	7563 (44.8)	549 (46.0)	593 (45.6)	83 (49.4)	6338 (44.6)
Race and ethnicity					
Hispanic	4342/16 667 (26.1)	335/1175 (28.5)	340/1284 (26.5)	55/167 (32.9)	3612/14 041 (25.7)
Non-Hispanic Black	7506/16 667 (45.0)	577/1175 (49.1)	670/1284 (52.2)	69/167 (41.3)	6190/14 041 (44.1)
Non-Hispanic White	3484/16 667 (20.9)	169/1175 (14.4)	186/1284 (14.5)	28/167 (16.8)	3101/14 041 (22.1)
Non-Hispanic other^f^	1335/16 667 (8.0)	94/1175 (8.0)	88/1284 (6.9)	15/167 (9.0)	1138/14 041 (8.1)
Influenza season					
2016-2017	5174 (30.7)	164 (13.7)	412 (31.7)	23 (13.7)	4575 (32.2)
2017-2018	4149 (24.6)	296 (24.8)	323 (24.8)	56 (33.3)	3474 (24.5)
2018-2019	4830 (28.6)	283 (23.7)	261 (20.1)	55 (32.7)	4231 (29.8)
2019-2020	2714 (16.1)	450 (37.7)	304 (23.4)	34 (20.2)	1926 (13.6)
Insurance status					
Private	2539/16 638 (15.3)	125/1174 (10.6)	149/1287 (11.6)	21/167 (12.6)	2244/14 010 (16.0)
Public	12 837/16 638 (77.2)	933/1174 (79.5)	1056/1287 (82.1)	134/167 (80.2)	10 714/14 010 (76.5)
Both	131/16 638 (0.8)	8/1174 (0.7)	6/1287 (0.5)	1/167 (0.6)	116/14 010 (0.8)
Self-pay	1131/16 638 (6.8)	108/1174 (9.2)	76/1287 (5.9)	11/167 (6.6)	936/14 010 (6.7)
Site					
Cincinnati, Ohio	2364 (14.0)	154 (12.9)	245 (18.8)	9 (5.4)	1956 (13.8)
Houston, Texas	1623 (9.6)	49 (4.1)	131 (10.1)	11 (6.5)	1432 (10.1)
Kansas City, Missouri	3129 (18.6)	206 (17.3)	231 (17.8)	13 (7.7)	2679 (18.9)
Nashville, Tennessee	3992 (23.7)	500 (41.9)	248 (19.1)	106 (63.1)	3138 (22.1)
Pittsburgh, Pennsylvania	2240 (13.3)	91 (7.6)	178 (13.7)	14 (8.3)	1957 (13.8)
Rochester, New York	1805 (10.7)	82 (6.9)	185 (14.2)	7 (4.2)	1531 (10.8)
Seattle, Washington	1714 (10.2)	111 (9.3)	82 (6.3)	8 (4.8)	1513 (10.7)
Symptom duration at encounter, median (IQR), d	3.0 (1.0-4.0)	2.0 (1.0-3.0)	3.0 (2.0-4.0)	2.0 (1.0-4.0)	3.0 (1.0-4.0)
Symptoms					
Cough only	4188/16 446 (25.5)	42/1192 (3.5)	100/1295 (7.7)	18/166 (10.8)	4028/13 793 (29.2)
Fever only	1834/16 446 (11.2)	70/1192 (5.9)	79/1295 (6.1)	16/166 (9.6)	1669/13 793 (12.1)
Fever and cough	10 424/16 446 (63.4)	1080/1192 (90.6)	1116/1295 (86.2)	132/166 (79.5)	8096/13 793 (58.7)
Influenza tested clinically					
Any clinical testing	3912 (23.2)	1026 (86.0)	223 (17.2)	108 (64.3)	2555 (18.0)
Rapid antigen	1786 (10.6)	532 (44.6)	130 (10.0)	100 (59.5)	1028 (7.2)
Rapid PCR	1095 (6.5)	372 (31.2)	44 (3.4)	5 (3.0)	680 (4.9)
Clinical PCR panel	1068 (6.3)	124 (10.4)	50 (3.8)	4 (2.4)	911 (6.4)
Influenza positive					
Any clinical testing	1104 (6.5)	1010 (84.7)	94 (7.2)	NA	NA
Rapid antigen	538 (3.2)	520 (43.6)	19 (1.5)	NA	NA
Rapid PCR	399 (2.4)	369 (30.9)	33 (1.7)	NA	NA
Clinical PCR panel	165 (0.1)	123 (10.3)	43 (2.3)	NA	NA
Research testing	2453 (14.5)	1164/1191 (97.7)	1289/1298 (99.3)	NA	NA
Research and clinical testing	1063 (6.3)	981 (82.2)	83 (6.4)	NA	NA
Positive for influenza and other respiratory virus	403 (2.4)	183 (15.3)	220 (16.9)	NA	NA
Positive for noninfluenza respiratory virus	9620 (57.0)	NA	NA	133 (79.2)	9529 (67.1)
Antiviral receipt or prescription during visit	863 (5.1)	616 (51.6)	85 (6.5)	62 (36.9)	101/14 203 (0.7)

Abbreviations: *ICD-10*, *International Statistical Classification of Diseases and Related Health Problems, Tenth Revision*; NA, not applicable; PCR, polymerase chain reaction.

^a^ Denominators are shown in cells where there are missing data for that subgroup.

^b^ True positive is defined as influenza positive and *ICD-10* positive.

^c^ False negative is defined as influenza positive and *ICD-10* negative.

^d^ False positive is defined as influenza negative and *ICD-10* positive.

^e^ True negative is defined as influenza negative and *ICD-10* negative.

^f^ Other race and ethnicity groups included American Indian or Alaska Native, Asian, Native Hawaiian or Other Pacific Islander, multiple, and none, consolidated because of small sample sizes.

**Table 2.** Demographic and Clinical Characteristics of Patients in the Inpatient Setting

Characteristic	Patients, No. (%)^a^				
	Total (N = 17 060)	True positive (n = 872)^b^	False negative (n = 371)^c^	False positive (n = 196)^d^	True negative (n = 15 621)^e^
Age at presentation, median (IQR), y	1.0 (0.0-4.0)	3.0 (1.0-8.0)	2.0 (0.0-7.0)	2.0 (0.0-6.0)	1.0 (0.0-4.0)
Age range at presentation, y					
0-1	9691 (56.8)	285 (32.7)	163 (43.9)	93 (47.4)	9150 (58.6)
2-4	3371 (19.8)	216 (24.8)	76 (20.5)	44 (22.4)	3035 (19.4)
5-17	3998 (23.4)	371 (42.5)	132 (35.6)	59 (30.1)	3436 (22.0)
Sex					
Male	9798 (57.4)	483 (55.4)	205 (55.3)	109 (55.6)	9001 (57.6)
Female	7262 (42.6)	389 (44.6)	166 (44.7)	87 (44.4)	6620 (42.4)
Race and ethnicity					
Hispanic	4035/16 911 (23.9)	195/865 (22.5)	58/369 (15.7)	66/192 (34.4)	3716/15 485 (24.0)
Non-Hispanic Black	4096/16 911 (24.2)	223/865 (25.8)	105/369 (28.5)	40/192 (20.8)	3728/15 485 (24.1)
Non-Hispanic White	7078/16 911 (41.9)	357/865 (41.3)	168/369 (45.5)	68/192 (35.4)	6485/15 485 (41.9)
Non-Hispanic other^f^	1702/16 911 (10.1)	90/865 (10.4)	38/369 (10.3)	18/192 (9.4)	1556/15 485 (10.0)
Study year					
2016-2017	4785 (28.0)	129 (14.8)	119 (32.1)	46 (23.5)	4491 (28.7)
2017-2018	4443 (26.0)	191 (21.9)	79 (21.3)	72 (36.7)	4101 (26.3)
2018-2019	4736 (27.8)	226 (25.9)	91 (24.5)	36 (18.4)	4383 (28.1)
2019-2020	3096 (18.1)	326 (37.4)	82 (22.1)	42 (21.4)	2646 (16.9)
Insurance status					
Private	5237/16 871 (31.0)	245/859 (28.5)	122/363 (33.6)	60/191 (31.4)	4810/15 458 (31.1)
Public	10 595/16 871 (62.8)	550/859 (64.0)	227/363 (62.5)	116/191 (60.7)	9702/15 458 (62.8)
Both	246/16 871 (1.5)	16/859 (1.9)	4/363 (1.1)	2/191 (1.0)	224/15 458 (1.4)
Self-pay	793/16 871 (4.7)	48/859 (5.6)	10/363 (2.8)	13/191 (6.8)	722/15 458 (4.7)
Site					
Cincinnati, Ohio	2205 (12.9)	81 (9.3)	52 (14.0)	24 (12.2)	2048 (13.1)
Houston, Texas	3679 (21.6)	174 (20.0)	47 (12.7)	52 (26.5)	3406 (21.8)
Kansas City, Missouri	1572 (9.2)	76 (8.7)	23 (6.2)	13 (6.6)	1460 (9.3)
Nashville, Tennessee	2396 (14.0)	133 (15.3)	45 (12.1)	66 (33.7)	2152 (13.8)
Pittsburgh, Pennsylvania	3888 (22.8)	240 (27.5)	160 (43.1)	29 (14.8)	3459 (22.1)
Rochester, New York	1841 (10.8)	71 (8.1)	36 (9.7)	7 (3.6)	1727 (11.1)
Seattle, Washington	1479 (8.7)	97 (11.1)	8 (2.2)	5 (2.6)	1369 (8.8)
Symptom duration at encounter, median (IQR), d	3.0 (2.0-5.0)	3.0 (2.0-5.0)	4.0 (2.0-6.0)	4.0 (3.0-6.0)	3.0 (2.0-5.0)
Symptoms					
Cough only	4911/16 674 (29.5)	40/867 (4.6)	48/369 (13.0)	20/194 (10.3)	4803/15 244 (31.5)
Fever only	1359/16 674 (8.2)	41/867 (4.7)	24/369 (6.5)	15/194 (7.7)	1279/15 244 (8.4)
Fever and cough	10 404/16 674 (62.4)	786/867 (90.7)	297/369 (80.5)	159/194 (82.0)	9162/15 244 (60.1)
Influenza tested clinically					
Any clinical testing	8233 (48.3)	651 (74.7)	165 (44.5)	67 (34.2)	7350 (47.1)
Rapid antigen	1117 (6.5)	138 (15.8)	43 (11.6)	43 (21.9)	893 (5.7)
Rapid PCR	1219 (7.1)	183 (21.0)	12 (3.2)	4 (2.0)	1020 (6.5)
Clinical PCR panel	6549 (38.4)	373 (42.8)	129 (34.8)	26 (13.3)	6021 (38.5)
Influenza positive					
Any clinical testing	722 (8.8)	643 (73.7)	79 (21.3)	NA	NA
Rapid antigen	150 (0.9)	134 (15.4)	16 (4.3)	NA	NA
Rapid PCR	186 (1.1)	183 (21.0)	3 (0.8)	NA	NA
Clinical PCR panel	437 (2.6)	369 (42.3)	68 (18.3)	NA	NA
Research testing	1120 (6.7)	778 (89.2)	342 (92.2)	NA	NA
Research and clinical testing	599 (3.6)	549 (63.0)	50 (13.5)	NA	NA
Positive for noninfluenza pathogen	11 281 (66.1)	NA	NA	123 (62.8)	11 158 (71.4)
Positive influenza and other respiratory pathogen	252 (1.5)	131 (15.0)	121 (32.6)	NA	NA
Influenza antiviral receipt or prescription during visit	1189 (7.0)	579 (66.4)	42 (11.3)	117 (59.7)	451/15 615 (2.9)
Hospital length of stay, median (IQR), d	2.0 (1.0-3.0)	2.0 (1.0-3.0)	1.0 (1.0-2.5)	2.0 (1.0-3.0)	2.0 (1.0-3.0)
Hospital length of stay <1 d	868 (5.1)	37 (4.2)	17 (4.6)	15 (7.7)	799 (5.1)

Abbreviations: *ICD-10*, *International Statistical Classification of Diseases and Related Health Problems, Tenth Revision*; NA, not applicable; PCR, polymerase chain reaction.

^a^ Denominators are shown in cells where there are missing data for that subgroup.

^b^ True positive is defined as influenza positive and *ICD-10* positive.

^c^ False negative is defined as influenza positive and *ICD-10* negative.

^d^ False positive is defined as influenza negative and *ICD-10* positive.

^e^ True negative is defined as influenza negative and *ICD-10* negative.

^f^ Other race and ethnicity groups included American Indian or Alaska Native, Asian, Native Hawaiian or Other Pacific Islander, multiple, and none, consolidated because of small sample sizes.

Compared with hospitalized children, children discharged from the ED were older, more frequently had public insurance, and more frequently received rapid antigen testing if tested. ED patients received clinical influenza testing and influenza antivirals less frequently than hospitalized patients. Overall, 3912 patients (23.2%) in the ED and 8233 patients (48.3%) in the hospital had clinical influenza testing. Among those with clinical testing, 52 patients were tested with antigen testing only (7 in the ED, and 45 in the hospital). Of those, 9 were positive (3 in the ED, and 6 in the hospital).

### ED Setting

In the ED, 2493 children (14.8%) tested positive for influenza by either research or clinical testing (40 [0.2%] clinical testing only, 1389 [8.2%] research testing only, and 1064 [6.3%] clinical and research testing), with 1193 true-positive and 1300 false-negative cases. Among those with a positive influenza clinical test, 94 (8.5%) did not receive an influenza diagnosis code. Those with both an *ICD-10* influenza diagnosis and laboratory-confirmed influenza (ie, true-positives) were older, had a shorter duration of illness onset to clinical presentation, and were more likely to have received an influenza antiviral medication compared with those with an *ICD-10* influenza diagnosis and negative influenza testing (ie, false-positives) (Table 1). The proportion of true-positive cases was lower during the 2016 to 2017 season compared with later seasons and highest at the Nashville study site.

### Inpatient Setting

For children admitted to the hospital, 1243 (7.5%) tested positive for influenza by either research or clinical testing (123 [0.7%] clinical testing only, 521 [3.1%] research testing only, and 599 [3.6%] clinical and research testing), with 872 true-positive and 371 false-negative cases (Table 2). Among those with a positive influenza clinical test, 79 (10.9%) did not receive an influenza diagnosis code. The proportion of true-positive cases was lowest in those aged 2 to 4 years and during the 2016 to 2017 season and highest at the Pittsburgh and Houston study sites. False-negative cases were more likely to be older, have rapid testing performed, have longer duration of illness onset to clinical presentation, and receive an antiviral during their encounter compared with true-negative cases. Among those hospitalized, 868 (5.1%) had a duration of hospitalization of 1 day or less.

### Accuracy of Influenza *ICD-10* Codes

In the ED, and across the NVSN influenza seasons, *ICD-10* influenza diagnoses were overall highly specific (98.0%; 95% CI, 97.8%-98.3%) with high PPV (88.6%; 95% CI, 88.0%-89.2%) and NPV (85.9%; 95% CI, 85.3%-86.6%), in contrast to their modest sensitivity (48.6%; 95% CI, 47.6%-49.5%) (Table 3). Among children in the inpatient setting, specificity was 98.2% (95% CI, 98.0%-98.5%) and PPV was 82.8% (95% CI, 82.1%-83.5%), whereas sensitivity was 70.7% (95% CI, 69.8%-71.5%) and NPV was 96.5% (95% CI, 96.2%-96.9%) (Table 4). PPV was associated with influenza prevalence, with the highest PPV occurring during periods of peak influenza activity (Figure, panel A).

**Table 3.** Accuracy of Influenza *ICD-10* Codes in the Emergency Department^a^

Variable	Percentage (95% CI)			
	Specificity	Sensitivity	Positive predictive value	Negative predictive value
Influenza *ICD-10 *code^b^	98.0 (97.8-98.3)	48.6 (47.6-49.5)	88.6 (88.0-89.2)	85.9 (85.3-86.6)
Age, y				
0-1	99.1 (98.9-99.3)	47.2 (46.2-48.3)	83.8 (83.0-84.6)	95.0 (94.5-95.5)
2-4	98.8 (98.5-99.1)	47.5 (46.1-48.9)	90.1 (89.2-90.9)	89.2 (88.3-90.0)
5-17	98.2 (97.7-98.6)	48.7 (47.1-50.4)	88.7 (87.7-89.7)	86.7 (85.6-87.8)
Alternate season definition				
Peak 13 wk	97.6 (97.3-98.0)	50.1 (48.9-51.4)	91.0 (90.4-91.7)	80.3 (79.3-81.2)
Peripeak 13 wk	98.5 (98.1-98.9)	42.2 (40.5-43.8)	69.6 (68.1-71.2)	95.4 (94.7-96.1)
Peak 33%	96.8 (96.0-97.5)	52.7 (50.6-54.9)	91.0 (89.8-92.2)	76.9 (75.1-78.6)
Prepeak 33%	99.0 (98.8-99.3)	41.1 (40.0-42.3)	85.4 (84.5-86.2)	92.3 (91.6-92.9)
Postpeak 33%	99.0 (98.8-99.3)	49.7 (48.6-50.8)	86.3 (85.6-87.1)	94.2 (93.7-94.7)
October to March	98.4 (98.1-98.6)	49.6 (48.7-50.5)	88.7 (88.1-89.3)	88.2 (87.6-88.8)

Abbreviation: *ICD-10*, *International Statistical Classification of Diseases and Related Health Problems, Tenth Revision*.

^a^ A total of 1193 patients were polymerase chain reaction (PCR) positive and *ICD-10* positive, 168 were PCR negative and *ICD-10* positive, 1300 were PCR positive and *ICD-10 negative*, and 14 206 were PCR negative and *ICD-10 *negative.

^b^ The New Vaccine Surveillance Network season is defined as the period spanning the first week with 2 positive influenza research tests through the last week with 2 positive influenza research tests for each surveillance site.

**Table 4.** Accuracy of Influenza *ICD-10* Codes in the Hospital^a^

Variable	Percentage (95% CI)			
	Specificity	Sensitivity	Positive predictive value	Negative predictive value
Influenza *ICD-10 *code^b^	98.2 (98.0-98.5)	70.7 (69.8-71.5)	82.8 (82.1-83.5)	96.5 (96.2-96.9)
Age, y				
0-1	99.0 (98.8-99.2)	63.6 (62.7-64.6)	75.4 (74.5-76.3)	98.3 (98.0-98.5)
2-4	98.6 (98.2-99.0)	74.0 (72.5-75.5)	83.1 (81.8-84.3)	97.6 (97.0-98.1)
5-17	98.3 (97.9-98.7)	73.8 (72.4-75.1)	86.3 (85.2-87.4)	96.3 (95.7-96.9)
Alternate season definition				
Peak 13 wk	97.8 (97.5-98.2)	73.6 (72.6-74.6)	84.7 (83.9-85.5)	95.8 (95.3-96.3)
Peripeak 13 wk	98.7 (98.3-99.1)	60.3 (58.7-62.0)	65.0 (63.3-66.6)	98.4 (98.0-98.9)
Peak 33%	97.5 (96.9-98.2)	76.3 (74.6-78.1)	86.3 (84.9-87.7)	95.3 (94.4-96.2)
Prepeak 33%	98.8 (98.5-99.0)	64.1 (63.0-65.2)	76.8 (75.8-77.8)	97.8 (97.4-98.1)
Postpeak 33%	99.1 (98.8-99.3)	70.6 (69.6-71.7)	82.1 (81.3-83.0)	98.2 (97.9-98.5)
October to March	98.4 (98.2-98.7)	71.7 (70.9-72.5)	82.6 (81.9-83.3)	97.1 (96.8-97.4)

Abbreviation: *ICD-10*, *International Statistical Classification of Diseases and Related Health Problems, Tenth Revision*.

^a^ A total of 872 patients were polymerase chain reaction (PCR) positive and *ICD-10* positive, 196 were PCR negative and *ICD-10* positive, 371 were PCR positive and *ICD-10 *negative, and 15 621 were PCR negative and *ICD-10 *negative.

^b^ The New Vaccine Surveillance Network season is defined as the period spanning the first week with 2 positive influenza research tests through the last week with 2 positive influenza research tests for each surveillance site.

**Figure.**
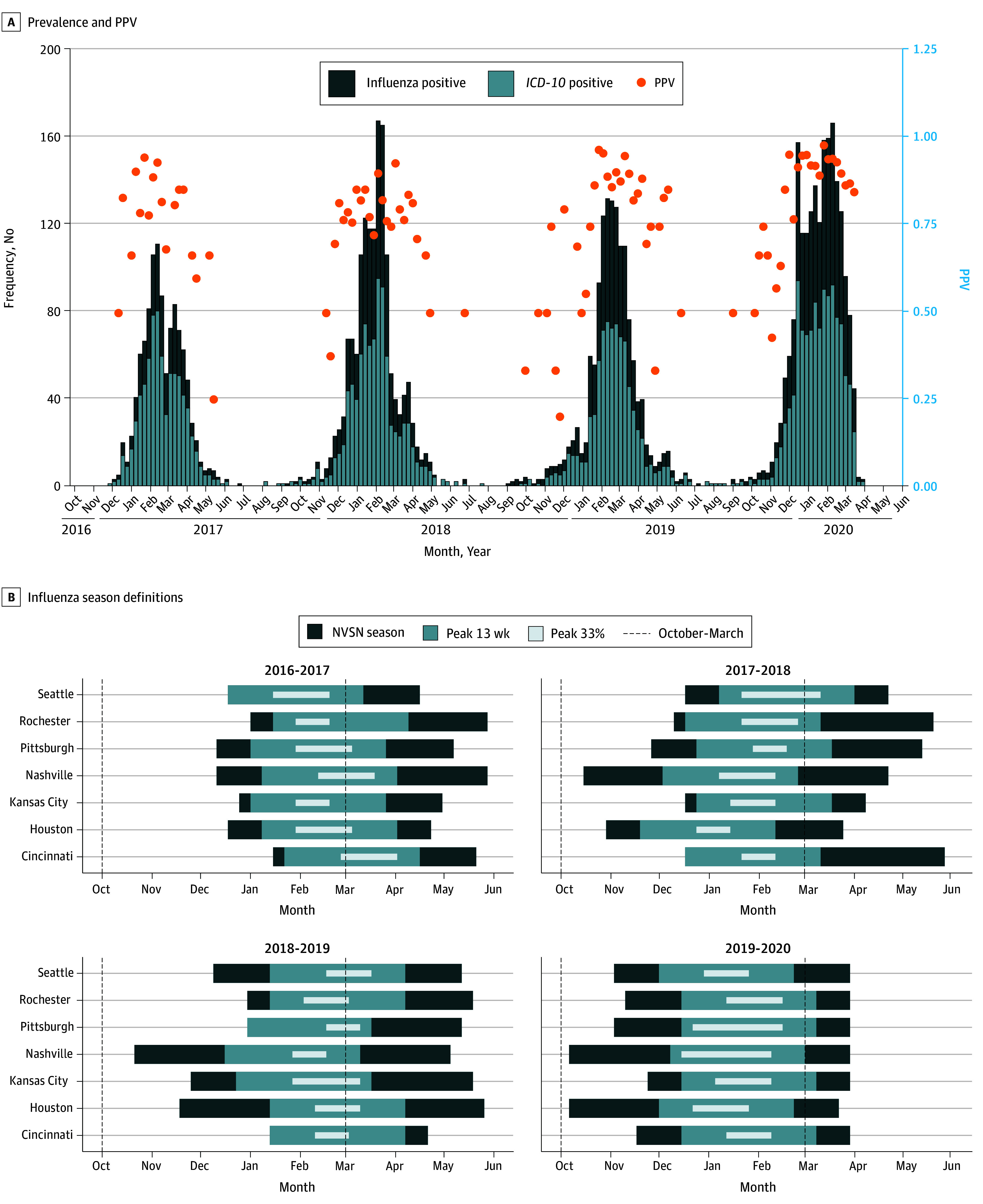
Prevalence and Positive Predictive Value (PPV) of Influenza Diagnoses and Influenza Season (A) Prevalence of influenza and PPV of *ICD-10* influenza diagnoses among children younger than 18 years. PPV could not be calculated during weeks when either influenza diagnosis or laboratory detections were zero. (B) Influenza season definitions. Dark blue bars indicate NVSN season definition (primary study period for analyses), defined as the period spanning the first through last weeks with 2 positive surveillance tests for each surveillance site. Medium blue bars indicate 13-week peak season definition, defined as the 13 consecutive weeks that contained the maximum number of influenza cases. Light blue bars indicate peak 33% season definition, defined as cases where onset of symptoms occurred after the date corresponding to the 33rd percentile and before or on the date corresponding to the 67th percentile of symptom onset date among enrolled influenza-positive cases. Vertical dashed lines represent October to March season definition. Note that not all sites and seasons using the NVSN definition consist of 13 weeks’ or longer duration.

### Age-Based Differences in *ICD-10* Code Accuracy

Specificity was similar across all age categories, whereas age differences were noted for sensitivity, PPV, and NPV (Table 3). PPV was lowest among children aged 0 to 1 year (ED, 83.8% [95% CI, 83.0%-84.6%]; inpatient, 75.4% [95% CI, 74.5%-76.3%]). In the ED, NPV was lowest among those aged 5 to 17 years (86.7%; 95% CI, 85.6%-87.8%). Similarly, in the inpatient setting, NPV was lowest among those aged 5 to 17 years (96.3%; 95% CI, 95.7%-96.9%).

### Alternative Diagnoses in Misclassified Influenza Cases

Among false-positive cases, the most common pathogens identified on clinical molecular or antigen testing were respiratory syncytial virus, rhinovirus and/or enterovirus, and adenovirus (eTable 1 in Supplement 1). Among false-negative cases, the most common pathogens identified other than influenza alone were influenza plus respiratory syncytial virus, influenza plus rhinovirus and/or enterovirus, and influenza plus adenovirus. The most common diagnoses among false-negative cases in the ED were other specified upper respiratory infection, fever, and respiratory signs and symptoms. The most common diagnoses among false-negative cases in the inpatient setting were fluid and electrolyte disorders, respiratory signs and symptoms, and other specified upper respiratory infections (eTable 2 in Supplement 1). Diagnoses and pathogens among misclassified cases were similar between ED and inpatient settings.

### Sensitivity Analyses

NPV increased when using a broader and more variable influenza season definition (October to March) and decreased when restricting to definitions incorporating weeks with higher influenza prevalence (peak 13 weeks and peak 33%) (Figure, panel B, Table 3, and Table 4). PPV was greater in both the ED and inpatient setting using the peak 13 weeks and peak 33% definitions compared with the seasonal definition used for our primary analysis (NVSN definition) and the October to March definition (Table 3 and Table 4).

When excluding individuals with antigen testing, both PPV and sensitivity decreased. Similarly, when excluding individuals with rapid testing (antigen or PCR), PPV and sensitivity decreased. The magnitude of effect was greater in the hospital than ED setting (eTable 3 in Supplement 1). When defining true influenza cases as those with a positive antigen or PCR test, PPV increased in both settings (ED, 90.5% [95% CI, 89.6%-91.4%]; inpatient, 91.1% [95% CI, 90.4%-91.7%]). When comparing time between onset and testing, PPV was higher when the time between onset and testing was 5 days or less (eTable 4 in Supplement 1). Defining a true influenza case as a positive clinical test increased the PPV in the hospital but not ED setting (eTable 5 in Supplement 1).

## Discussion

Using data from a large, active, prospective, multicenter study during 4 influenza seasons from 2016 to 2020 and involving more than 32 000 children in either the ED or inpatient setting, this cohort study found that influenza *ICD-10* codes were moderately sensitive and highly specific for identifying cases of laboratory-confirmed influenza. Importantly, influenza *ICD-10* codes had both high PPV and NPV, with PPV being greatest in peak seasons. We evaluated different influenza season definitions that may be used in future studies, depending on the prioritization of higher PPV or NPV. In studies using data without access to laboratory testing results, the peak 13 week or peak 33% season definitions likely reduce misclassification of the influenza diagnosis compared with the October to March definition. Performance of influenza *ICD-10* codes varied according to age and care setting. Our study provides guidance for the use of *ICD-10* influenza diagnosis codes for influenza surveillance and epidemiologic studies when laboratory confirmation is not available.

Our findings have major implications for the design and interpretation of future studies evaluating influenza. First, for observational studies that compare influenza and noninfluenza cases of respiratory infection, influenza *ICD-10* codes may be a valid and efficient method of case identification because of their high PPV for laboratory-confirmed influenza. Second, for studies of influenza disease burden, our findings of moderate sensitivity of influenza *ICD-10* codes mean that using these codes to identify cases of influenza illness will miss approximately one-half of cases in the ED and one-third of cases in the inpatient setting. There were generally low rates of influenza testing (23.2% in the ED and 48.3% in the hospital); furthermore, among patients with a positive influenza clinical test, 8.5% of patients in the ED and 10.9% inpatients did not receive an influenza diagnosis code. Estimates of influenza disease burden based on case identification with *ICD-10* diagnosis codes may need to be adjusted for underascertainment.^21,22^ Furthermore, the accuracy of *ICD-10* diagnoses is associated with clinical testing. With increasing availability of influenza testing and changes in testing practices following the COVID-19 pandemic, the accuracy of influenza diagnoses may increase in the future. Third, our results may inform the design of future age-based analyses. We identified age-based differences in the PPVs of influenza *ICD-10* diagnosis codes, with the lowest PPV among hospitalized children younger than 1 year despite similar proportion of clinical testing compared with other ages. It is possible that the prevalence of other circulating viruses known to cause respiratory illness in young children may affect pathogen-specific physician coding. Future studies are needed to improve identification of hospitalized infants with influenza when using electronic health record or administrative claims databases.

Our finding of differences in the accuracy of influenza *ICD-10* codes between ED and inpatient settings should be taken in context. Influenza diagnoses in the ED had lower sensitivity compared with those in the hospital. This difference is likely attributed, in part, to differences in encounter time, testing modality, and frequency of clinical testing. ED visits are shorter than hospitalizations, and although more rapid testing is performed in the ED, there are likely a notable number of patients who are discharged from the ED before the testing result notification. We also found that PPV was lower in the inpatient setting compared with the ED, and the reasons for this are likely multifactorial. Given that time since symptom onset to specimen collection for hospitalized patients was longer than for those in the ED, it is possible that there were more false-negative testing results in the hospital compared with the ED among those who would have tested positive earlier in their course of illness. Furthermore, nonrapid PCR testing was used significantly more in the hospital compared with ED setting. Given that 5.1% of hospitalization stays were 1 day or shorter, it is possible that patients were discharged from the hospital before the clinician received the test results. This is supported by findings of lower PPV in both the hospital and ED settings when excluding rapid testing.

### Limitations

This study has several limitations. We conducted prospective surveillance across multiple influenza seasons at 7 geographically and demographically diverse pediatric medical centers. Testing and/or coding practices at these medical centers may not be generalizable to other settings, and these data predate the COVID-19 pandemic. Misclassification of laboratory-confirmed influenza case status may have occurred, although we used molecular laboratory testing, which is generally considered the reference standard for identifying influenza.

## Conclusions

The findings of this study suggest that influenza *ICD-10* diagnoses are highly specific in identifying laboratory-confirmed influenza in the ED and inpatient settings, but their sensitivity is modest. *ICD-10* diagnosis codes performed best during periods with higher influenza prevalence, highlighting the influence of seasonal definitions on studies that use *ICD-10* diagnosis codes for influenza case identification. These findings may aid interpretation of studies that use *ICD-10* diagnosis codes to identify cases of influenza illness.

## Notes

Supplement 1. **eAppendix.** Supplemental Methods **eTable 1.** Respiratory Viruses Identified on Clinical Testing Among False Positive and False Negative Cases **eTable 2.** Most Common *ICD-10* Diagnoses Among False Negative Cases **eTable 3.** Sensitivity Analyses Restricting Clinical Testing Type **eTable 4.** Sensitivity Analyses Stratifying by Laboratory Tests < 5 Days and > 5 Days From Disease Onset **eTable 5.** Sensitivity Analyses Defining a True Influenza Case as a Positive Clinical Test **eFigure.** Flow Diagram

Supplement 2.
Data Sharing Statement
