# Supplementary material for: Accuracy of Influenza ICD-10 Diagnosis Codes in Identifying Influenza Illness in Children
Source: JAMA Netw Open. 2024 Apr 24;7(4):e248255. doi: 10.1001/jamanetworkopen.2024.8255 (PMC11043895; doi:10.1001/jamanetworkopen.2024.8255)
Supplement: Supplement 1. — eAppendix. Supplemental Methods eTable 1. Respiratory Viruses Identified on Clinical Testing Among False Positive and False Negative Cases eTable 2. Most Common ICD-10 Diagnoses Among False Negative Cases eTable 3. Sensitivity Analyses Restricting Clinical Testing Type eTable 4. Sensitivity Analyses Stratifying by Laboratory Tests < 5 Days and > 5 Days From Disease Onset eTable 5. Sensitivity Analyses Defining a True Influenza Case as a Positive Clinical Test eFigure. Flow Diagram [file jamanetwopen-e248255-s001.pdf]

Supplemental Online Content

Antoon JW, Stopczynski T, Amarin JZ, et al. Accuracy of influenza *ICD-10* diagnosis codes in identifying influenza illness in children. *JAMA Netw Open*. 2024;7(4):e248255. doi:10.1001/jamanetworkopen.2024.8255

eAppendix. Supplemental Methods

eTable 1. Respiratory Viruses Identified on Clinical Testing Among False Positive and False Negative Cases

eTable 2. Most Common *ICD-10* Diagnoses Among False Negative Cases

eTable 3. Sensitivity Analyses Restricting Clinical Testing Type

eTable 4. Sensitivity Analyses Stratifying by Laboratory Tests < 5 Days and > 5 Days From Disease Onset

eTable 5. Sensitivity Analyses Defining a True Influenza Case as a Positive Clinical Test

eFigure. Flow Diagram

This supplemental material has been provided by the authors to give readers additional information about their work.

© 2024 Antoon JW et al. *JAMA Network Open.*

eAppendix. Supplemental Methods

Influenza ICD-10 codes: J09, J09X, J09X1, J09X2, J09X3, J09X9, J10, J100, J1001, J1008, J101, J102, J108, J1081, J1082, J1083, J1089, J11, J110, J1100, J1108, J111, J112, J118, J1181, J1182, J1183, J1189

Diagnostic Testing: Diagnostic assay methods varied by site and included Luminex NxTAG Respiratory Pathogen Panel (Cincinnati and Kansas City), BioFire FilmArray Respiratory Panel (Seattle), Applied Biosystems TaqMan Array Microfluidic Card (Rochester), and in-house RT-PCR assays (Houston, Pittsburgh, and Nashville).[18] All laboratory sites passed CDC-sponsored proficiency testing.[18,19]

© 2024 Antoon JW et al. *JAMA Network Open.*

**eTable 1.** Respiratory Viruses Identified on Clinical Testing Among False Positive and False Negative Cases							
**False Positive Cases**				**False Negative Cases**			
**ED, n=168**	**n (%)**	**Inpatient, n=196**	**n (%)**	**ED, n=1,300**	**n (%)**	**Inpatient, n=371**	**n (%)**
RSV	22 (13.1)	RSV	35 (17.9)	Flu	1,080 (83.1)	Flu	250 (67.4)
RV/EV	17 (10.1)	RV/EV	22 (11.2)	RV/EV/Flu	74 (5.7)	RSV/Flu	41 (11.1)
AdV	16 (9.5)	hMPV	13 (6.6)	AdV/Flu	39 (3.0)	RV/EV/Flu	25 (6.7)
hMPV	9 (5.4)	PIV	11 (5.6)	RSV/Flu	35 (2.7)	CoV/Flu	9 (2.4)
PIV	9 (5.4)	AdV	5 (2.6)	CoV/Flu	28 (2.2)	hMPV/Flu	8 (2.2)
CoV	7 (4.2)	RSV/AdV	4 (2.0)	hMPV/Flu	8 (0.6)	AdV/Flu	8 (2.2)
RSV/AdV	3 (1.8)	RSV/CoV	4 (2.0)	PIV/Flu	6 (0.5)	RSV/RV/ EV/Flu	6 (1.6)
RV/EV/AdV	2 (1.2)	RSV/RV/EV	3 (1.5)	RV/EV/AdV/Flu	4 (0.3)	PIV/Flu	4 (1.2)
RV/EV/hMPV	2 (1.2)	CoV	2 (1.0)	RSV/CoV/Flu	3 (0.2)	AdV/CoV/Flu	3 (0.8)
RV/EV/PIV	2 (1.2)	RSV/PIV	2 (1.0)	RSV/RV/EV/ CoV/Flu	3 (0.2)	RSV/AdV/Flu	3 (0.8)
				RSV/RV/EV/Flu	3 (0.2)	AdV/ hMPV/Flu	2 (0.5)

Abbreviations: RSV, respiratory syncytial virus; RV, rhinovirus; EV, enterovirus; Adv, adenovirus; CoV, coronavirus; hMPV, Human metapneumovirus; PIV, parainfluenza virus

© 2024 Antoon JW et al. *JAMA Network Open.*

eTable 2. Most Common *ICD-10* Diagnoses Among False Negative Cases[a]

ED	n (%) N=2,520	Inpatient	n (%) N=1,485
Other specified upper respiratory infections	531 (21.1)	Fluid and electrolyte disorders	121 (8.1)
Fever	414 (16.4)	Respiratory signs and symptoms	95 (6.4)
Respiratory signs and symptoms	336 (13.3)	Other specified upper respiratory infections	94 (6.3)
Otitis media	209 (8.3)	Asthma	93 (6.3)
Viral infection	203 (8.1)	Acute bronchitis	84 (5.7)
Nausea and vomiting	91 (3.6)	Pneumonia	63 (4.2)
Asthma	76 (3.0)	Fever	59 (4.0)
Abdominal pain and other digestive/abdomen signs and symptoms	62 (2.5)	Other general signs and symptoms	44 (3.0)
Other general signs and symptoms	59 (2.3)	Epilepsy; convulsions	43 (2.9)

[a]May include >1 diagnosis per patient.

© 2024 Antoon JW et al. *JAMA Network Open.*

eTable 3. Sensitivity Analyses Restricting Clinical Testing Type

	Excluding individuals with antigen testing				Excluding individuals with rapid testing (PCR or antigen)				Defining influenza cases as a positive antigen or PCR test			
	**ED**		**Hospital**		**ED**		**Hospital**		**ED**		**Hospital**	
	**PCR +**	**PCR -**	**PCR +**	**PCR -**	**PCR +**	**PCR -**	**PCR +**	**PCR -**	**PCR +**	**PCR -**	**PCR +**	**PCR -**
ICD 10 +	661	68	734	151	289	63	551	147	1026	108	651	64
ICD 10 -	1170	13159	328	14579	1126	12480	316	13564	223	2533	165	7168
Accuracy measure												
PPV	90.7 (90.2, 91.1)		82.9 (82.4, 83.5)		82.1 (81.5, 82.7)		78.9 (78.3, 79.6)		90.5 (89.6, 91.4)		91.1 (90.4, 91.7)	
NPV	91.8 (91.4, 92.3)		97.8 (97.6, 98.0)		91.7 (91.3, 92.2)		97.7 (97.5, 98.0)		91.9 (91.1, 92.8)		97.8 (97.4, 98.1)	
Sensitivity	36.1 (35.3, 36.9)		69.1 (68.4, 69.8)		20.4 (19.8, 21.1)		63.6 (62.8, 64.3)		82.2 (80.9, 83.4)		79.8 (78.9, 80.7)	
Specificity	99.5 (99.4, 99.6)		99.0 (98.8, 99.1)		99.5 (99.4, 99.6)		98.9 (98.8, 99.1)		95.9 (95.3, 96.5)		99.1 (98.9, 99.3)	

© 2024 Antoon JW et al. *JAMA Network Open.*

eTable 4. Sensitivity Analyses Stratifying by Laboratory Tests ≤ 5 Days and > 5 Days From Disease Onset

	**ED**				**Hospital**			
	≤ 5 days from disease onset		> 5 days from disease onset		≤ 5 days from disease onset		> 5 days from disease onset	
	**PCR +**	**PCR -**	**PCR +**	**PCR -**	**PCR +**	**PCR -**	**PCR +**	**PCR -**
ICD 10 +	1,092	150	100	18	672	122	199	74
ICD 10 -	1,083	11,761	216	2,432	271	12,326	99	3,277
Accuracy measure								
PPV	87.9 (87.4, 88.5)		84.8 (83.4, 86.1)		84.6 (84.0, 85.3)		72.9 (71.5, 74.3)	
NPV	91.6 (91.1, 92.0)		91.8 (90.8, 92.9)		97.9 (97.6, 98.1)		97.1 (96.5, 97.6)	
Sensitivity	50.2 (49.4, 51.0)		31.7 (29.9, 33.4)		71.3 (70.5, 72.0)		66.8 (65.3, 68.3)	
Specificity	98.7 (98.6, 98.9)		99.3 (99.0, 99.6)		99.0 (98.9, 99.2)		97.8 (97.3, 98.3)	

© 2024 Antoon JW et al. *JAMA Network Open.*

eTable 5. Sensitivity Analyses Defining a True Influenza Case as a Positive Clinical Test

	ED		Hospital	
	**PCR +**	**PCR -**	**PCR +**	**PCR -**
ICD 10 +	1,010	124	643	75
ICD 10 -	94	2,684	79	7,436
Accuracy measure				
PPV	89.1 (88.1, 90.0)		89.6 (88.9, 90.2)	
NPV	96.6 (96.0, 97.2)		98.9 (98.7, 99.2)	
Sensitivity	91.5 (90.6, 92.4)		89.1 (88.4, 89.7)	
Specificity	95.6 (94.9, 96.2)		99.0 (98.8, 99.2)	

© 2024 Antoon JW et al. *JAMA Network Open.*

eFigure. Flow Diagram

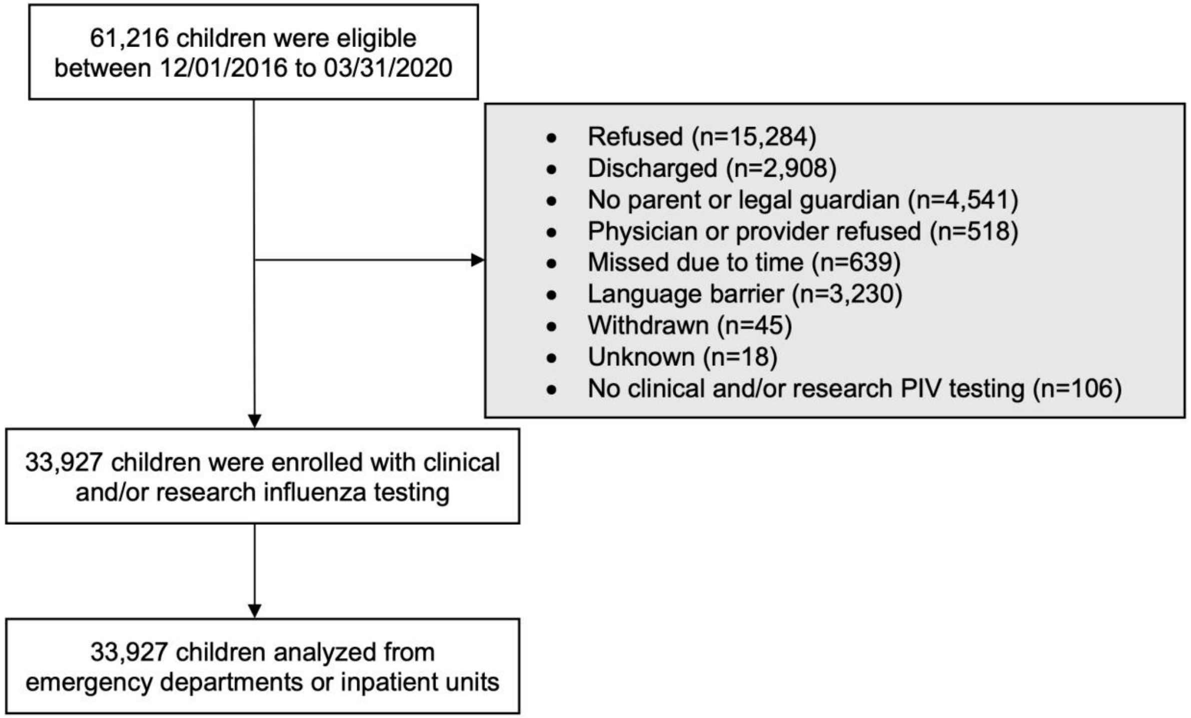

© 2024 Antoon JW et al. *JAMA Network Open.*
