# Supplementary material for: Accuracy of Influenza ICD-10 Diagnosis Codes in Identifying Influenza Illness in Children
Source: JAMA Netw Open. 2024 Apr 24;7(4):e248255. doi: 10.1001/jamanetworkopen.2024.8255 (PMC11043895; doi:10.1001/jamanetworkopen.2024.8255)
Supplement: Supplement 2. — Data Sharing Statement [file jamanetwopen-e248255-s002.pdf]

Data Sharing Statement

Antoon. Accuracy of Influenza ICD-10 Diagnosis Codes in Identifying Influenza Illness in Children. *JAMA Netw Open*. Published April 24, 2024.
doi:10.1001/jamanetworkopen.2024.8255

Data
Data available: No

Additional Information
Explanation for why data not available: The data that support the findings of this study are available from the corresponding author upon reasonable request. The data are not publicly available due to privacy or ethical restrictions. Leila Sahni and Samantha Olson had full access to all of the data in the study and take responsibility for the integrity and the accuracy of the data analysis.
